# The C-tail anchored TssL subunit, an essential protein of the enteroaggregative *Escherichia coli* Sci-1 Type VI secretion system, is inserted by YidC

**DOI:** 10.1002/mbo3.9

**Published:** 2012-03

**Authors:** Marie-Stéphanie Aschtgen, Abdelrahim Zoued, Roland Lloubès, Laure Journet, Eric Cascales

**Affiliations:** Laboratoire d'Ingénierie des Systèmes Macromoléculaires, Institut de Microbiologie de la Méditerranée, Aix-Marseille UniversitéCNRS – UMR 7255, 31 chemin Joseph Aiguier, 13402, Marseille Cedex 20, France

**Keywords:** Hcp, inner membrane, insertion, protein trafficking, topology

## Abstract

Type VI secretion systems (T6SS) are macromolecular complexes present in Gram-negative bacteria. T6SS are structurally similar to the bacteriophage cell-puncturing device and have been shown to mediate bacteria–host or bacteria–bacteria interactions. T6SS assemble from 13 to 20 proteins. In enteroaggregative *Escherichia coli* (EAEC), one of the subassemblies is composed of four proteins that form a trans-envelope complex: the TssJ outer membrane lipoprotein, the peptidoglycan-anchored inner membrane TagL protein, and two putative inner membrane proteins, TssL and TssM. In this study, we characterized the TssL protein of the EAEC Sci-1 T6SS in terms of localization, topology, and function. TssL is a critical component of the T6SS, anchored to the inner membrane through a single transmembrane segment located at the extreme C-terminus of the protein. We further show that this transmembrane segment is essential for the function of the protein and its proper insertion in the inner membrane is dependent upon YidC and modulated by the Hsp70 homologue DnaK.

## Introduction

Type VI secretion systems (T6SS) are macromolecular systems widely distributed in Gram-negative bacteria. Although T6SS have been first described as involved in pathogenesis, recent data demonstrated that T6SS participate in the adaptation to the environment by regulating biofilm formation or killing neighburing bacteria (Bingle et al. 2008; Jani and Cotter 2010; Hood et al. 2010; Schwarz et al. 2010; Russell et al. 2011). Recent progresses have been made regarding the physiological roles of T6SS and the regulatory mechanisms controlling their expression and production (Cascales 2008; Bernard et al. 2010; Leung et al. 2011); however, how these macromolecular complexes assemble in the bacterial cell envelope remains elusive.

T6SS are composed of at least 13 components (called “core-components”), named Tss (Type VI secretion; Shalom et al. 2007), but the number of subunits encoded by most T6SS gene clusters varies up to 25 (reviewed in references Boyer et al. 2009, Bingle et al. 2008; Cascales 2008; Filloux et al. 2008). Only a few subunits have been functionally or structurally characterized (Cascales and Cambillau 2012). Several of these proteins share similarities with bacteriophage T4 tail and baseplate components, and it has been suggested that part of T6SS might derive from bacteriophage. The crystal structures of Hcp and VgrG have been reported: Hcp forms hexameric rings leaving a pore of ∼40 Å (Mougous et al. 2006; Jobichen et al. 2010) whereas three VgrG assemble to form a needle-like structure (Pukatzki et al. 2007, 2009; Leiman et al. 2009). The Hcp structure is superimposable to the major tail protein gpV of bacteriophage λ (corresponding to the bacteriophage T4 gp19 protein; Pell et al. 2009) whereas VgrG has a fold highly similar to the gp27/gp5 complex, the cell puncturing device of bacteriophage T4 (Kanamaru et al. 2002; Pukatzki et al. 2007; Leiman et al. 2009). The TssE subunit has structural similarities with gp25, a component of the bacteriophage baseplate (Cascales 2008; Lossi et al. 2011). The *Vibrio cholerae* TssB (VipA) and TssC (VipB) proteins have been shown to form tubular structures resembling the bacteriophage T4 sheath (Bönemann et al. 2009; Cascales and Cambillau 2012), which can be disassembled by ClpV, an AAA^+^ ATPase (Bönemann et al. 2009, 2010; Pietrosiuk et al. 2011).

Aside from bacteriophage-derived components, a number of membrane-associated proteins are associated with T6SS. At least three proteins—TssJ, TssL, and TssM—form a trans-envelope complex, that may be augmented by TagL, an additional protein containing a peptidoglycan-binding domain (Aschtgen et al. 2010a). TssJ is an outer membrane lipoprotein (Aschtgen et al. 2008) whose structure has been reported recently (Felisberto-Rodrigues et al. 2011). TssJ interacts with the inner membrane TssM subunit, an IcmF-like protein (Zheng and Leung 2007; Felisberto-Rodrigues et al. 2011). TssM interacts with TssL, an IcmH-like protein (Zheng and Leung 2007; Ma et al. 2009). IcmF and IcmH are two components required for the optimal function of Type IVb secretion systems in *Legionella pneumophila* and *Coxiella burnetii* (Segal et al. 2005; Nagai and Kubori 2011). The T6SS TssL and T4bSS IcmH proteins are closely related (DUF2077 family). Although the enteroaggregative *Escherichia coli* (EAEC) TssL and the *L. pneumophila* IcmH proteins share 45% of similarity, they endow conserved secondary structure predictions (Fig. S1). TagL is a polytopic inner membrane protein shown to interact directly with TssL (Aschtgen et al. 2010a). The periplasmic domain of TagL carries a peptidoglycan-binding motif of the OmpA/Pal/MotB family (pfam PF05691; Aschtgen et al. 2010a, 2010b) that presumably anchors the T6SS to the cell wall.

Herein, we further characterized the TssJLM–TagL complex of the EAEC Sci-1 T6SS focusing on the TssL subunit (GenBank accession number CBG37349; locus tag EC042_4527). We report that TssL is an essential protein for the function of the Sci-1 apparatus and demonstrate that TssL is an inner membrane protein. Further topology experiments using cysteine and protease accessibility assays showed that TssL is anchored to the inner membrane through a transmembrane domain located at the extreme C-terminus of the protein. This topology being unusual we tested the genetic requirements for TssL insertion in the inner membrane. Our data suggest that proper insertion of TssL is Tat independent, Sec independent and signal recognition particle (SRP) independent but requires the YidC protein and is modulated by the DnaK chaperone.

## Experimental Procedures

### Bacterial strains, media, growth conditions, and chemicals

Bacteria strains are listed in Table S1. *E. coli* K12 DH5α was used for cloning procedures. The enteroaggregative *E. coli* strain 17-2 (kindly provided by Arlette Darfeuille-Michaud, University of Clermont-Ferrand, France) and its Δ*tssL* (Δ*sciP*; Aschtgen et al. 2010a) derivative were used for this study. *Escherichia coli* K12 MC4100 *secB*::Cm^r^, Δ*tig*::Cm^r^, *dnaK-dnaJ*::Kan^r^, *dnaJ*::Tn*10*-42 (Tet^r^), and *dnaJ*::Tn*10*-42 *djlA*ΩSpc^r^ (Kang and Craig 1990; Genevaux et al. 2001; Ullers et al. 2007) and the *yidC* conditional mutant strain (FTL10, an MC4100 *yidC*-deleted strain carrying a chromosomal copy of the *yidC* gene under the control of the arabinose-inducible P*araBAD* promoter; Hatzixanthis et al. 2003) were kindly provided by Pierre Genevaux (LMGM, Toulouse, France). The FtsY-depletion strain (IY28 [Erez et al. 2010]) was kindly provided by Hans-Georg Koch (Freiburg Universität, Germany). The *tatABCDE* (DADE, Wexler et al. 2000) and the conditional temperature-sensitive *secA* (MM52, Oliver and Beckwith 1981) strains were kindly provided by Long-Fei Wu (LCB, Marseille, France). Except the conditional *yidC* and *ftsY* mutants, strains were routinely grown in LB (Luria Broth) broth at 37°C, with aeration. The YidC- and FtsY-depletion strains were grown overnight in LB medium supplemented with L-arabinose 0.5% and then diluted in fresh LB medium without arabinose and cultured for 3 h before induction of *tssL*. The *secA* MM52 strain was grown at 28°C for 3 h before *tssL* induction. Expression of *tssL* (from plasmid pIBA-TssL) was obtained by addition of anhydrotetracyclin (AHT). For the Hcp release, fractionation, selective solubilization, and cysteine and protease accessibility assays, the *sci-1* gene cluster was induced by addition of the iron chelator 2,2′′-dipyridyl (125 μM final concentration) 30 min prior harvesting the cells (Brunet et al. 2011). Plasmids and mutant alleles were maintained by the addition of ampicillin (100 μg mL^−1^ for *E. coli* K12, 200 μg mL^−1^ for EAEC), kanamycin (50 μg mL^−1^ for *E. coli* K12, 50 μg mL^−1^ for chromosomal insertion on EAEC, 100 μg mL^−1^ for plasmid-bearing EAEC), chloramphenicol (40 μg mL^−1^), or tetracycline (12 μg mL^−1^). Sodium lauroyl sarcosinate (SLS), L-arabinose, *N*-ethyl-maleimide (NEM), and yeast carboxypeptidase Y (CPY) were purchased from Sigma-Aldrich (Saint-Quentin Falavier, France), AHT (used at 0.2 μg mL^−1^ throughout the study) from IBA, proteinase K from Merck, and 3-(*N*-maleimidyl-propionyl) biocytin (MPB) from Molecular probes. Anti-TolB, anti-TolR, anti-OmpA, and anti-OmpF polyclonal antibodies are from the laboratory collection. The anti-AcrA polyclonal antibody has been kindly provided by Muriel Masi (IBBMC, Paris Sud, France). Anti-EFTu (mAb900 clone, Hycult Biotech), anti- hemagglutinine (HA) (3F10 clone, Roche), and anti-FLAG (M2 clone, Sigma-Aldrich) monoclonal antibodies are commercially available.

### Plasmid construction

Polymerase chain reactions (PCR) were performed with a Biometra thermocycler, using the Pfu Turbo DNA polymerase (Stratagene, La Jolla, California). Custom oligonucleotides were synthesized by Eurogentec and are listed in Table S1. TssL-producing plasmids have been constructed by a double PCR technique, allowing amplification of the gene of interest flanked by extensions annealing to the target vector (van den Ent and Löwe 2006; Aschtgen et al. 2010a). The product of the first PCR has then been used as primers for a second PCR using the target vector as template. pIBA-TssL encodes the full-length TssL protein carrying a N-terminal FLAG tag cloned into the pASK-IBA37(+) vector (IBA) using oligonucleotides IBA-TssL-5 and IBA-TssL-3. pIBA-TssL-Lt encodes the full-length TssL carrying a N-terminal FLAG tag and a C-terminal Lumio-tag (CCPGCC) extension cloned into the pASK-IBA37(+) vector using oligonucleotides IBA-TssL-5 and IBA-TssL-Lt-3. Construction of the pIBA-TssL-PG plasmid, encoding the full length TssL protein fused to the periplasmic domain of the TagL protein, will be described elsewhere. Construction of pIBA-TssL-ΔTM has been constructed by site-directed mutagenesis using pIBA-TssL as template and oligonucleotides TssL-Y184St-5 and TssL-Y184St-3 (complementary oligonucleotides introducing a codon stop at the Y184 codon [TAC modified as TAA]). All constructs have been verified by restriction analyses and DNA sequencing (Genome Express).

### Biofilm formation

The adherence assay was performed in 24-well polystyrene microtitre dishes, glass tubes, and cover-glasses, after incubation at 30°C without agitation for 20 h. Attached bacteria were stained with 1% crystal violet for 15 min and washed four times with water. For quantification, the ring of stained bacteria was collected with 500 μl of 95% ethanol and diluted in the same volume of water. The absorbance of the suspension was then measured at 590 nm. This value was divided by the absorbance of the bacterial culture (measured at 600 nm) and data are reported relative to the wild-type strain.

### Hcp release assay

Supernatant and cell fractions have been separated as previously described (Aschtgen et al. 2010a) except that the iron chelator 2,2′-dipyridyl (125 μM final concentration) was added to the culture medium 30 min before harvesting the cells (Brunet et al. 2011). Briefly, 2 × 10^9^ cells producing HA epitope-tagged Hcp (from plasmid pOK-HcpHA; Aschtgen et al. 2010a) were harvested and collected by centrifugation at 2000 × *g* for 5 min. The supernatant fraction was then subjected to a second low-speed centrifugation and then at 16,000 × *g* for 15 min. The supernatant was filtered on sterile polyester membranes with a pore size of 0.2 μm (membrex 25 PET, membraPure GmbH) before precipitation with trichloroacetic acid (TCA) 15%. Cells and precipitated supernatant were resuspended in loading buffer and analyzed by Sodium Dodecyl Sulfate-Polyacrylamide Gel Electrophoresis (SDS-PAGE) and immunoblotting with the anti-HA antibody. As control for cell lysis, Western blots were probed with antibodies raised against the periplasmic TolB protein.

### Fractionation

A total of 2 × 10^9^ exponentially growing cells were resuspended in 1 mL of 10 mM Tris-HCl (pH 8.0), sucrose 20%, and incubated for 10 min on ice. After addition of 100 μg mL^−1^ of lysozyme and 0.5 mM Ethylene diamino tetra-acetic acid (EDTA) and further incubation for 45 min on ice, DNase (100 μg mL^−1^) and MgCl_2_ (2 mM) were added and cells were lysed by five cycles of freeze and thaw. Unbroken cells were removed by centrifugation, and soluble and membrane fractions were separated by ultracentrifugation for 40 min at 100,000 × *g*. Membranes were washed with 20 mM Tris-HCl pH8.0, MgCl_2_ 2 mM, and resuspended in 1 mL of 20 mM Tris-HCl, 150 mM NaCl, 8 M urea, incubated on a wheel for 1 h. The mixture was then ultracentrifuged for 40 min at 100,000 × *g* to separate integral membrane and peripherally membrane-associated proteins. Soluble and membrane-associated fractions were precipitated with TCA (15%), and resuspended in loading buffer prior to analyze by SDS-PAGE and immunoblotting.

### SLS extraction

SLS is an anionic detergent that selectively disrupts the inner membrane and solubilizes inner membrane proteins (Filip et al. 1973). Membranes prepared from 10^10^ cells using the fractionation protocol were resuspended in 1 mL of 10 mM Tris-HCl (pH 8.0), EDTA 1 mM supplemented with 1% of sodium *N*-lauroyl sarcosinate (SLS, Sigma-Aldrich) and incubated on a wheel for 1 h at room temperature. Insoluble (outer membrane) and soluble (inner membrane) fractions were collected by ultracentrifugation at 100,000 × *g* for 40 min before analysis by SDS-PAGE and immunoblotting.

### Sedimentation sucrose gradient

Inner and outer membranes were separated using discontinuous sedimentation sucrose gradients. A total of 2 × 10^11^ cells were harvested, resuspended in 1.5 mL of Tris-HCl 10 mM pH7.4, sucrose 20%, and RNase 100 μgmL^−1^, lysed by French-press treatment (four passages at 1200 psi), and total membranes were recovered by centrifugation at 100,000 × *g* for 40 min and resuspended in 0.5 mL of 20% sucrose containing a protease inhibitor cocktail (Complete EDTA-free, Roche). The membrane fraction was then loaded on the top of a discontinuous sucrose gradient composed of the superposition of 1.5 mL of 30, 35, 40, 45, 50, 55, and 60% sucrose solutions (from top to bottom). Gradients were centrifuged at 90,000 × *g* for 90 h and 500 μl fractions were collected from the top. The fractions were analyzed by a Nicotinamide Adenine Dinucleotide (NADH) oxidase enzymatic test, and by SDS-PAGE and immunoblotting with the anti-AcrA (inner membrane) and anti-OmpF (outer membrane) antibodies. The NADH oxidase activity was measured in 96-well polystyrene microtitre dishes, using 20 μl of each fraction diluted in 180 μl of Tris-HCl 50 mM pH7.5, dithiothreitol (DTT) 0.2 mM, and NADH 0.5 mM. The decrease of absorbance of the NADH at 340 nm, which reflects the activity of the NADH oxidase, was measured after 15 min of incubation at 25°C using a TECAN M200 microplate reader. Each fraction has been tested in duplicate.

### Cysteine accessibility experiments (SCAM)

Cysteine accessibility experiments (Bogdanov et al. 2005) were carried out as described by Jakubowski et al. (2004) and Goemaere et al. (2007) with modifications. A 40-mL culture of strain Δ*tssL* producing wild-type TssL or TssL fused to a C-terminal lumio tag (CCPGCC) was induced for *tssL* gene expression with 0.2 μg mL^−1^ AHT for 2 h. Cells were harvested, resuspended in buffer A (100 mM Hepes [pH 7.5], 250 mM sucrose, 25 mM MgCl_2_, 0.1 mM KCl) to a final optical density at 600 nm (OD_600_) of 12 in 500 μl of buffer A. 3-(*N*-) MPB (Molecular Probes) was added to a final concentration of 100 μM (from a 20 mM stock freshly dissolved in Dimethyl Sulfoxide (DMSO)) and the cells were incubated for 30 min at 25°C. β-Mercaptoethanol (20 mM final concentration) was added to quench the biotinylation reaction, and cells were washed twice in buffer A, and resuspended in buffer A containing *N*-ethyl maleimide (final concentration 5 mM) to block all free sulfhydryl residues. After incubation for 20 min at 25°C, cells were disrupted by sonication. Membranes recovered by ultracentifugation for 40 min at 100,000 × *g* were resuspended in 1 mL of buffer B (10 mM Tris (pH 8.0), 100 mM NaCl, 1% (v/v) Triton X-100, protease inhibitor cocktail [Complete, Roche]). After incubation on a wheel for 1 h, unsolubilized material was removed by centrifugation for 15 min at 20,000 × *g*, and solubilized proteins were subjected to immunoprecipitation using FLAG M2 beads (Sigma-Aldrich). After 3 h of incubation on a wheel, beads were washed twice with 1-mL buffer B, once with buffer B supplemented with Tween 0.1%, and once with buffer C (10 mM Tris [pH 8.0], 100 mM NaCl, 0.1% [w/v] Triton X-100). Beads were air-dried and resuspended in Laemmli buffer prior to SDS-PAGE analysis and immunodetection with anti-FLAG antibodies (to detect the TssL proteins) or streptavidin (to detect the biotinylated TssL proteins) coupled to alkaline phosphatase.

### Protease accessibility experiments

A total of 2 × 10^10^ exponentially growing cells were harvested on ice for 15 min, pelleted by centrifugation (whole cells sample), and suspended in 250 μl spheroplast buffer (100 mM Tris-HCl, pH 8.0, sucrose 30% w/v). Lysozyme was added to the final concentration of 100 μg mL^−1^, followed by the addition of 250 μl of 100 mM Tris-HCl, EDTA 2 mM, pH 8.0. The cells were gently mixed and then incubated for 10 min at 4°C. The spheroplasts were pelleted by centrifugation at 2000 × *g* for 10 min and resuspended in 50 μl of 100 mM Tris-HCl, sucrose 15%, pH 8.0. As control, whole cells or whole membranes were resuspended in 50 μl of 100 mM Tris-HCl, sucrose 15%, pH 8.0. proteinase K (Merck) was then added in each sample to the final concentration of 250 μg mL^−1^, and the mixtures were further incubated for 30 min on ice. For CPY accessibility experiments, whole membranes were resuspended in 50 μl of CPY assay buffer (MES 25 mM, NaCl 50 mM, pH 6.0). CPY (Sigma-Aldrich) was added to the final concentration of 250 μg mL^−1^ and the samples were further incubated for 2 h at 30°C. Proteolysis was quenched by the addition of an equal volume of 2 × Laemmli loading buffer.

### In vivo TssL insertion assay

The insertion of TssL into the inner membrane was tested as described (Fontaine et al. 2001) with modifications. Expression of the *tssL* gene cloned into the AHT-inducible pASK-IBA37(+) vector was induced in wild-type and mutant strains, as well as in conditional mutant strains under restrictive conditions. About 2 × 10^10^ cells were harvested, pelleted by centrifugation, and resuspended in 1 mL of 50 mM Tris-HCl, pH 8.0, EDTA 1 mM, urea 8 M. After 1 h incubation on a wheel, cells were disrupted by sonication. Whole cells were discarded by low-speed centrifugation, and membranes were pelleted by ultra-speed centrifugation for 45 min at 100,000 × *g*. Proteins of the soluble fractions were TCA-precipitated. Inner membrane proteins were extracted from the membrane pellet using SLS 0.5% in Tris-HCl, pH 8.0, EDTA 1 mM. Proteins of the supernatant fraction (inner membrane proteins) were TCA-precipitated. Both soluble and inner membrane fractions were resuspended in Laemmli loading buffer prior to SDS-PAGE analysis and immunodetection.

## Results

### *tssL* encodes a protein essential for Type VI secretion

**Figure 1:**
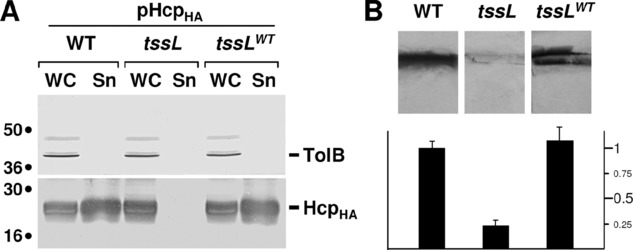
The TssL protein is required for Type VI secretion. (A) Effect of the *tssL* mutation on Hcp protein release. Hcp_HA_ release was assessed by separating whole cells (WC) and supernatant (Sn) fractions from WT, *tssL*, or complemented *tssL* (*tssL*^WT^) cultures. A total of 2 × 10^8^ cells and the TCA-precipitated material of the supernatant from 5 × 10^8^ cells were subjected to 12.5% acrylamide SDS-PAGE and immunodetected using the anti-HA monoclonal antibody (lower panel) and the anti-TolB polyclonal antibodies (lysis control; upper panel). (B) Effect of the *tssL* mutation on biofilm formation. Biofilms formed in static cultures of WT, *tssL* or complemented *tssL* (*tssL*^WT^) cells were visualized on cover glass by crystal violet staining (upper panel) and quantified using the ethanol-solubilization procedure, relative to the WT EAEC strain (lower graph).

To gain insight on the role of the TssL protein of the Sci-1 EAEC T6SS, we first constructed a strain deleted of the *tssL* gene. To test whether TssL is required for Type VI secretion, we used the biofilm and Hcp release assays as reporters, two assays previously reported to reflect the correct assembly of the Sci-1 T6SS (Aschtgen et al. 2008). As shown in Figure 1, the *tssL* gene is an essential component of Type VI secretion machines as *tssL* cells (i) did not release Hcp in the culture supernatant (Fig. 1A) and (ii) were severely impaired in their capacity to form biofilm (Fig. 1B). Complementation experiments using a plasmid encoding TssL fused to an N-terminal Flag epitope showed that production of the epitope-tagged TssL protein restored Hcp release and biofilm formation to wild-type (WT) levels. This demonstrates that the epitope-tagged TssL protein is functional and that the *tssL* deletion has no polar effect on downstream genes. It is noteworthy that the *aaiN* gene encoding the *tssL* homologue of cluster *sci-2* does not compensate for the loss of *tssL*, suggesting that no cross-talk occurs between *aaiN* and *tssL* in EAEC.

### TssL is an integral inner membrane protein

**Figure 2:**
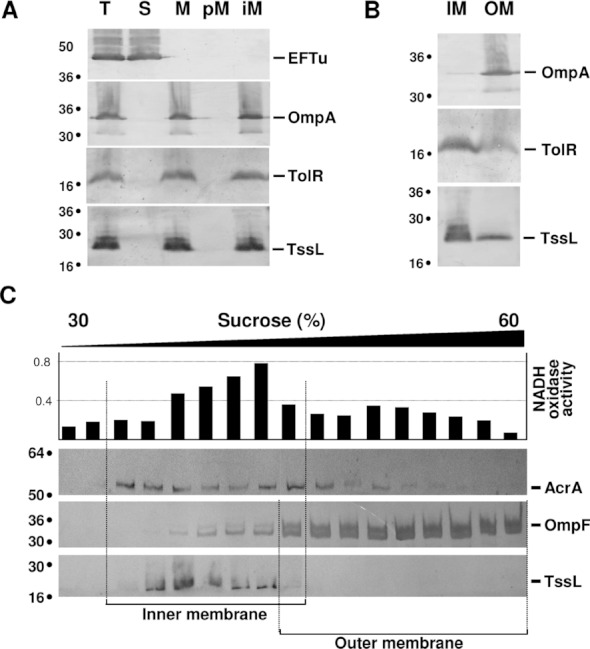
TssL is an integral inner membrane protein. (A) TssL co-fractionates with integral membrane proteins. A fractionation procedure was applied to EAEC cells producing _Flag_TssL (T, total fraction), allowing separation between the soluble (S) and membrane (M) fractions. Membranes were then treated with urea to separate peripheral membrane (pM) and integral membrane (iM) proteins. Samples were subjected to 12.5% acrylamide SDS-PAGE and immunodetected with antibodies directed against the EFTu (soluble), TolR (integral inner membrane), and OmpA (integral outer membrane) proteins, and the Flag epitope of TssL. (B) Total membranes from EAEC cells producing _Flag_TssL (T) were treated with SLS, and solubilized inner membrane (IM) and insolubilized outer membrane (OM) proteins were separated. Samples from 5 × 10^8^ cells were subjected to 12.5% acrylamide SDS-PAGE and immunodetected with antibodies directed against the TolR (inner membrane), OmpA (outer membrane) proteins, and against the Flag epitope of TssL. (C) Total membranes (T) from EAEC cells producing _Flag_TssL were separated on a discontinuous sedimentation sucrose gradient. Collected fractions were analyzed for contents using the anti-AcrA (inner membrane), anti-OmpF (outer membrane), and anti-Flag antibodies, and with a NADH oxidase (inner membrane) activity test (upper graph). Although AcrA is an integral IM protein, its distribution in the sucrose gradient extends to the OM fractions due to its interaction with the OM protein TolC. The positions of the inner and outer membrane-containing fractions are indicated. Molecular weight markers are indicated on the left of each panel.

We further tested TssL subcellular localization using fractionation experiments. As shown in Figure 2A, TssL was immunodetected in the membrane fraction. Further experiments showed that TssL was not released from the membranes upon urea (Fig. 2A) or sodium carbonate treatment (data not shown), demonstrating that TssL is an integral membrane protein. Selective procedures allowing specific solubilization of inner membrane proteins using further showed that TssL was solubilized in presence of SLS. Although a portion of the TssL protein remained insoluble, the profile of TssL solubilization is comparable to that of the TolR inner membrane protein, thus suggesting that TssL locates in the inner membrane (Fig. 2B). This result was confirmed by sedimentation gradient experiments: TssL co-fractionated with inner membrane proteins such as AcrA or the NADH oxidase, but did no co-fractionate with higher density fractions containing the outer membrane protein OmpF (Fig. 2C). Taken together, these data show that TssL is an integral protein of the inner membrane.

### TssL is anchored through a single C-terminal transmembrane segment

**Figure 3:**
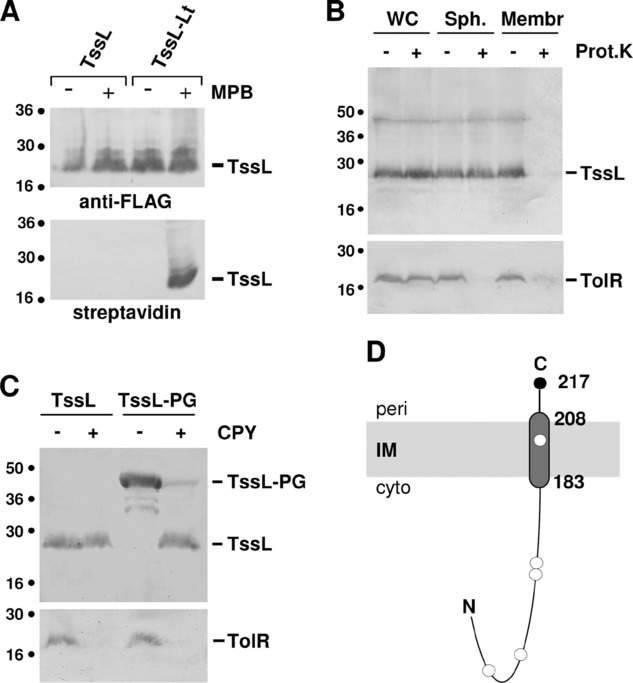
Topology of the EAEC TssL protein. (A) Accessibility of cysteine residues. Whole EAEC *tssL* cells producing the _Flag_TssL or _Flag_TssL-Lt (_Flag_TssL carrying a C-terminal CCPGCC motif) were treated (+) or not (−) with the 3-(*N*-)MPB probe, solubilized, and the TssL proteins were precipitated using agarose beads coupled to M2 anti-FLAG antibody. Precipitated material was subjected to SDS-PAGE and Western blot analysis using anti-FLAG antibody (to detect TssL, upper panel) and streptavidin coupled to alkaline phosphatase (to detect biotinylated TssL, lower panel). Molecular weight markers are indicated on the left. (B) Accessibility of TssL to proteinase K. Whole cells (WC), spheroplasts (Sph.), or purified membranes (Membr.) of *tssL* EAEC cells producing the _Flag_TssL protein were treated (+) or not (−) with proteinase K and subjected to SDS-PAGE and Western-blot analyses using anti-TolR (an in-to-out topology inner membrane protein with the bulk of the protein in the periplasm, lower panel) and anti-FLAG (upper panel) antibodies. (C) Accessibility of TssL to CPY. Purified membranes of *tssL* EAEC cells producing the _Flag_TssL protein (TssL) or a _Flag_TssL protein fused to the 211-amino acid periplasmic domain of TagL (TssL-PG) were treated (+) or not (−) with CPY and subjected to SDS-PAGE and Western-blot analyses using anti-TolR (lower panel) and anti-FLAG (upper panel) antibodies. (D) Topology model for the TssL protein at the inner membrane. The positions of the labeled (C-terminus) and unlabeled (C40, C68, C127, C129, and C200) cysteine residues are indicated by filled and open circles, respectively. The membrane boundaries of the transmembrane segment predicted by computer algorithms and identified by the accessibility studies are indicated.

The EAEC Sci-1 TssL protein is composed of 217 amino acids. Computer analyses predict the existence of a single transmembrane (TM) segment located between residues 183 and 208, suggesting that TssL is a bitopic protein. However, while most computer programs predict an out-to-in topology (N-terminus located in the periplasm) a few predict an in-to-out topology (N-terminus located in the cytoplasm). To experimentally define the TssL topology and discriminate between these two hypotheses, we first performed a cysteine accessibility assay using SCAM (substituted cysteine accessibility method; Bogdanov et al. 2005). This assay relies on the fact that the *N*-maleimide biocytin (MPB) probe readily passes the outer membrane but is unable to cross the inner membrane. Therefore, only cysteine residues located in the periplasm can be labeled. TssL possesses five cysteine residues, one located within the putative TM segment (at position 200), and four located within the N-terminal soluble domain (at positions 40, 68, 127, and 129). Figure 3A showed that the full-length TssL protein was not biotinylated, demonstrating that none of the native TssL cysteine residues were accessible to the *N*-MPB probe (Fig. 3A). This suggests that the native cysteines locate in the cytoplasm or are buried within the structure. However, MPB treatment of membranes containing TssL showed that TssL was labeled demonstrating that at least one of the cysteine residues in the N-terminal region is not buried. This result therefore suggests that the TssL N-terminus is located in the cytoplasm. To confirm this topology, we added a lumio-tag (or FLASH-tag), constituted of four cysteine residues (CCPGCC), at the C-terminus of the protein. The TssL derivative (TssL-Lt) was shown to be functional as it complemented the *tssL* mutant strain in the Hcp release and biofilm formation assays (data not shown). SCAM experiments showed that TssL-Lt was labeled with MPB, demonstrating that the C-terminal lumio tag resides in the periplasm (Fig. 3A). Identical results were obtained in the EAEC pathotype and in a laboratory *E. coli* K12 strain (i.e., devoid of T6SS gene cluster) producing TssL, demonstrating that TssL topology is independent on other T6SS subunits (data not shown).

This topology, determined by the cysteine substitution approach, was confirmed by protease accessibility experiments. First, we tested TssL accessibility to proteinase K. As shown in Figure 3B, the full-length TssL protein was not degraded when whole cells or spheroplasts were treated with proteinase K, whereas it was sensitive to proteinase K in membrane preparations. This result suggests that the N-terminal soluble bulk of the protein locates in the cytoplasm. As control, the in-to-out bitopic TolR inner membrane protein, for which the soluble domain is exposed to the periplasm (Muller et al. 1993), was degraded in both intact and lysed spheroplasts. To further validate the SCAM and proteinase K accessibility experiments, we tested TssL accessibility to CPY, a protease degrading proteins from their C-terminus. Using the CPY degradation assay, the TolR protein was fully degraded in membrane preparations (Fig. 3C). Conversely, the TssL protein was resistant to the action of CPY in membrane preparations, suggesting that the C-terminus of TssL is not accessible to CPY or that CPY degradation is arrested by the transmembrane segment (Fig. 3C). Therefore, we constructed a TssL protein carrying a C-terminal extension of 211 amino acids corresponding to the periplasmic domain of the TagL protein. This extended TssL protein (predicted size ∼ 47 kDa) was degraded to a ∼ 23 kDa fragment corresponding to the full-length TssL protein upon treatment of membrane preparations with CPY. This result suggests that the C-terminal extension of the extended TssL protein was accessible to CPY and that CPY degradation was terminated at the inner membrane. All together, the cysteine accessibility and protease degradation data demonstrate that TssL is a bitopic membrane protein with an in-to-out topology as schematized in Figure 3D. The transmembrane segment being located at the extreme C-terminus (amino acids 183–208), the soluble domain therefore protrudes into the cytoplasm.

### The transmembrane domain of TssL is required for T6SS function

**Figure 4:**
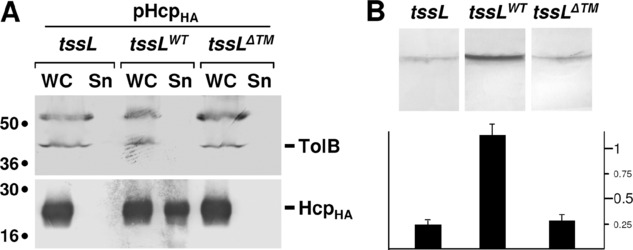
TssL transmembrane anchor is required for function. (A) Hcp protein release. Hcp_HA_ release (produced from plasmid pHcp_HA_) was assessed by separating whole cells (WC) and supernatant (Sn) fractions from cultures of *tssL*, or *tssL* cells producing WT TssL (*tssL*^WT^) or TssL deleted of its C-terminal transmembrane anchor (*tssL*^ΔTM^). A total of 2 × 10^8^ cells and the TCA-precipitated material of the supernatant from 5 × 10^8^ cells were subjected to 12.5% acrylamide SDS-PAGE and immunodetected using the anti-HA monoclonal antibody (lower panel) and the anti-TolB polyclonal antibodies (lysis control; upper panel). (B) Biofilm formation. Biofilms formed in static cultures of *tssL*, *tssL*^WT^, or *tssL*^ΔTM^ cells were visualized on cover glass by crystal violet staining (upper panel) and quantified using the ethanol-solubilization procedure, relative to the WT EAEC strain (lower graph).

Our previous results demonstrated that TssL is anchored to the inner membrane through a C-terminal transmembrane segment. To determine if TssL anchorage is required for the assembly of the T6SS, we introduced a stop codon at position 184, a few residues before the transmembrane segment (amino acids 189–211). This construct, called TssL-ΔTM, therefore lacks the TM segment as well as six residues in the periplasm. Fractionation data showed that TssL-ΔTM co-fractionate with the EFTu cytoplasmic protein (data not shown). Complementation assays using the Hcp release and biofilm formation assays as reporters showed that TssL-ΔTM was not able to complement the *tssL* mutant, even though TssL-ΔTM was produced at similar levels compared to the full-length TssL (Fig. 4). The anchorage of TssL to the inner membrane is therefore required for a functional assembly of the T6SS.

### Membrane insertion of TssL is dependent on YidC

**Figure 5:**
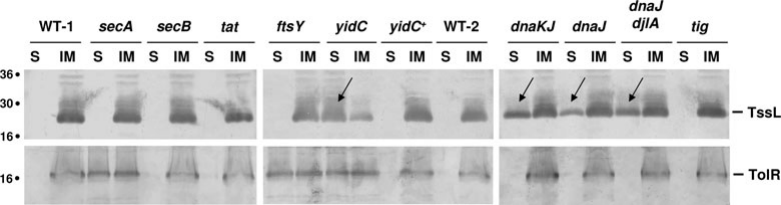
TssL insertion is dependent upon YidC and DnaK. Soluble proteins (soluble and peripherally associated membrane proteins; S) and integral inner membrane proteins (IM) were collected from various genetic backgrounds (WT-1, MC4100; *secA*, MM52 grown in restrictive conditions; *secB*, A443; *tat*, DADE; *ftsY*, IY28 grown in restrictive conditions; *yidC*, FTL10 grown in restrictive conditions; *yidC^+^*, FTL10 grown in permissive conditions; WT-2, BW25113; *dnaKJ*, PK101; *dnaJ*, GP108; *dnaJ djlA*, GP110; *tig*, A1091) and subjected to 12.5% acrylamide SDS-PAGE. The localization of the TolR and _Flag_TssL proteins was assessed by immunodetection using anti-TolR and anti-FLAG antibodies, respectively. Arrows indicate soluble fractions of strains in which the TssL protein is not properly inserted in the inner membrane. Molecular weight markers are indicated on the left.

This topology in which a cytoplasmic N-terminal domain is anchored through a transmembrane domain located at the extreme C-terminus is relatively uncommon in prokaryotic membrane proteins. In silico analyses recently suggested that only 11 proteins of *E. coli* K12 are predicted to have such a topology (Borgese and Righi 2010). These proteins, called C-tailed anchored (CTA) proteins, therefore, may represent as little as 0.2% of the total proteins in *E. coli*. Because the C-terminal anchor emerges from the ribosome upon translation termination, the insertion of these proteins in the inner membrane should follow specific rules, and may require additional partners to proceed such as chaperone or insertion catalyzers. By comparison with eukaryotic CTA proteins for which the insertion is better documented and has been shown to be dependent—in most cases—of Hsp70, it has been suggested that prokaryotic TA proteins might be dependent on the bacterial Hsp70 homologue, DnaK (Borgese and Righi 2010). We then tested TssL inner membrane insertion in various mutant backgrounds, including *secA* (the general Sec export pathway), *secB* (the chaperone of the Sec system), *tig* (the trigger factor that assists folding of nascent polypeptides emerging from the ribosome), *ftsY* (the membrane receptor of the SRP), *yidC* (an inner membrane insertase acting dependently or independently of the Sec and/or SRP machineries), *dnaK-dnaJ* (the Hsp70 homologue and its co-chaperone DnaJ), and *dnaJ-djlA* (the DnaK co-chaperone DnaJ and a membrane-anchored DnaJ paralogue). TssL production was induced during restrictive conditions and cells were harvested and disrupted by sonication in presence of urea. The supernatant contained soluble proteins as well as peripherally associated membrane proteins. Inner membranes were solubilized in presence of SLS and collected upon ultra-centrifugation. The TolR protein, which has an opposite topology (in-to-out bitopic protein with a large domain in the periplasm) was used as a control. As reported for this topology (van der Laan et al. 2001; Wang and Dalbey 2011), TolR insertion required SRP, the Sec translocon, as well as the YidC insertase (Fig. 5). Regarding TssL, our results show that its insertion at the inner membrane is dependent upon the YidC protein (Fig. 5). We did not observe TssL mis-localization in Sec-deficient (*secA*, *secB*) or SRP-deficient (*ftsY*) strains. Although less pronounced, a significant and reproducible amount of TssL was found in the soluble fraction of the *dnaK* or *dnaJ* mutant strains (Fig. 5). These data suggest that TssL insertion is catalyzed by YidC and modulated by the Hsp70 homologue DnaK.

## Discussion

In the work described in this manuscript, we characterized one of the core-components of the T6SS, the TssL subunit. *tssL* in one of the 13 genes strictly conserved in all T6SS gene clusters (Boyer et al. 2009). As expected, we first showed that TssL is essential for the function of the EAEC Sci-1 T6SS. Homologues of TssL have already been shown to be indispensable for T6S in *Edwardsiella tarda* (Zheng and Leung 2007), *Agrobacterium tumefaciens* (Ma et al. 2009), *V. cholerae* (Zheng et al. 2011) and *Francisella tularensis* (de Bruin et al. 2011). TssL is closely related to the IcmH/DotU proteins associated with Type IVb secretion systems in *L. pneumophila* and *Coxiella burnetti* (Segal et al. 2005; Nagai and Kubori 2011). Contrary to essential components of the *L. pneumophila* T4bS translocon, *icmH* is not essential for the function of the secretion system but is rather required for the optimal efficiency of substrate translocation, Dot/Icm-mediated plasmid transfer and intracellular replication in macrophage (Sexton et al. 2004; vanRheenen et al. 2004; Zusman et al. 2004).

Using a combination of fractionation, selective solubilization, sedimentation gradients, and cysteine and protease accessibility experiments, we determined that TssL is an in-to-out bitopic inner membrane protein. The TssL protein adopts identical localization and topology in EAEC and *E. coli* K12, suggesting that none of the T6SS components controls its characteristics. These localization and topology are compatible with a recent report showing similar characteristics for the *A. tumefaciens* ImpK TssL homologue (Ma et al. 2009). However, the *Agrobacterium* ImpK protein carries an additional domain at the C-terminus. This domain is predicted to have a peptidoglycan binding activity and is homologue to the TagL periplasmic domain. Therefore, ImpK is an “evolved” TssL protein, resulting from the fusion of TssL and a peptidoglycan-binding motif (Ma et al. 2009; Aschtgen et al. 2010b). The IcmH/DotU protein of the *L. pneumophila* T4bSS has been shown to be an integral membrane protein (Sexton et al. 2004), but its topology has not been yet experimentally determined. However, vanRheenen et al. reported that the *L. pneumophila* IcmH protein is non accessible to an inner membrane-impermeant biotinylated molecule, suggesting that the protein is mostly exposed in the cytoplasm (vanRheenen et al. 2004). Indeed, computer predictions and topology experiments of the EAEC TssL protein showed that the bulk of the protein localizes in the cytoplasm and that the TM segment is located between residues 183 and 208, leaving a short 9-residue C-terminal fragment in the periplasm. Because of the sequence similarities between the TssL and the IcmH/DotU proteins, we propose that these proteins share a common topology. This membrane configuration is quite uncommon, as only 11 proteins from *E. coli* K12 are predicted to have C-tail anchors (Borgese and Righi 2010). By analogy with the insertion of eukaryotic C-tail anchored proteins it has been suggested that the insertion of prokaryotic CTA proteins might be dependent on the DnaKJ chaperone system (Borgese and Righi 2010); however, although a small portion was not properly inserted, the majority of TssL is still anchored in the inner membrane of *E. coli* K12 *dnaKJ* cells (Fig. 5). This result might be due to overlapping chaperone functions in *E. coli*. We also tested the efficiency of TssL insertion in various mutant strains, including most genes described as involved in inner membrane biogenesis. Our results suggest that TssL insertion is Tat- and Sec-independent but rather requires the YidC insertion catalyzer. The YidC protein supports the insertion of Sec-independent inner membrane proteins, including proteins with different topologies (see Kol et al. 2008; Xie and Dalbey 2008; Kuhn 2009; Dalbey et al. 2011; Wang and Dalbey 2011). Proteins might be targeted to YidC by the SRP pathway, but our results of TssL localization in the *ftsY* conditional mutant strain, the gene encoding the SRP membrane receptor, showed that TssL is inserted in an SRP-independent manner. As a whole, our results suggest that TssL is therefore inserted by a YidC-only pathway and supported by the cytoplasmic chaperone DnaK. However, it is noteworthy that a small amount of TssL remains tightly associated with the inner membrane in absence of YidC, leaving open the hypothesis that an additional mechanism facilitates TssL insertion. It is noteworthy that the TssL TM segment (MYWLSWGAGIVTLAGLWCVLSSVLA) has a moderate hydrophobicity. It remains therefore possible that higher hydrophobicity CTA proteins are inserted through a different mechanism. Future work by testing the in vitro insertion of the TssL protein into reconstituted liposomes, or by assessing insertion of additional CTA proteins, will greatly help to better understand CTA proteins biogenesis.

Hence, the TssL protein might be divided into two functional domains, the cytoplasmic region and the TM segment. What can be the roles of these domains? One may hypothesize that the TM segment is involved in complex formation with the polytopic TssM protein, an homologue of the *L. pneumophila* IcmF protein, which forms a stable complex with IcmH (Sexton et al. 2004; vanRheenen et al. 2004). Indeed, TssL and TssM have been shown to interact in *E. tarda* and *A. tumefaciens* (Zheng and Leung 2007; Ma et al. 2009). In *L. pneumophila*, the IcmF-H complex stabilizes other components of the secretion apparatus (Sexton et al. 2004). A similar but more essential function might be suggested for the TssL-TssM complex. An alternative function for the TssL-M complex is the formation of a translocon through the inner membrane: as the Hcp and VgrG proteins are lacking Sec and Tat-dependent signal peptides but are found in culture supernatants and accumulate in the periplasm (Aschtgen et al. 2008; Jobichen et al. 2010), the TssL-M complex might be required for the passage of these two proteins into the periplasm and/or outside of the cell. In this case, an attractive hypothesis will be that the cytoplasmic domain of TssL might act as a cytoplasmic hook to recruit these proteins or protein effectors to the apparatus.

## Supporting Information

**Table S1.** Strains, plasmids, and oligonucleotides used in this study.

**Figure S1.** Sequence alignment of the IcmH protein from Legionella pneumophila str. Philadelphia (accession number YP_094503) and of the TssL protein from enteroaggregative Escherichia coli (accession number CBG37349).
