# Supplementary material for: The C-tail anchored TssL subunit, an essential protein of the enteroaggregative Escherichia coli Sci-1 Type VI secretion system, is inserted by YidC
Source: Microbiologyopen. 2012 Mar;1(1):71–82. doi: 10.1002/mbo3.9 (PMC3426401; doi:10.1002/mbo3.9)
Supplement: Supplementary file 1 [file mbo30001-0071-SD1.doc]

**Supplementary Table S1. Strains, plasmids and oligonucleotides used in this study.**

**Strains description and genotype source**

Enteroaggregative *E. coli*

17-2 WT enteroaggregative *Escherichia coli* Arlette Darfeuille-Michaud

17-2Δ*tssL* 17-2 deleted of the *sci1 tssL* gene Aschtgen *et al*., 2010a

*E. coli* K12

DH5α F-, Δ(*argF*-*lac*)U169, *phoA*, *supE44*, Δ(lacZ)M15, *relA*, *endA*, *thi*, *hsdR* Laboratory collection

MC4100 F-, Δ(*argF-lac*)U169, *araD139*, *rpsL150*, *relA1*, *deoC*, *rbs*, *flbB5301* Laboratory collection

MM52 (*secA*) MC4100 *secA-*ts Oliver and Beckwith, 1981

DADE (*tat*) MC4100 Δ*tatABCDE* Wexler *et al*., 2000

FTL10 (*yidC*) MC4100 *yidC*, *attB*::(*araC* P*araBAD*-*yidC*), Kan^r^ Hatzixanthis *et al*., 2003

A443 (*secB*) MC4100 *secB*::Cm^r^ Ullers *et al*., 2007

PK101 (*dnaKJ*) MC4100 *dnaK*, *dnaJ*::Kan^r^ Kang and Craig, 1990

GP108 (*dnaJ*) MC4100 dnaJ::Tn10-42(Tet^r^) Genevaux *et al*., 2001

A1091 (*tig*) MC4100 Δ*tig*::Cm^r^ Ullers *et al*., 2007

GP110 (*dnaJ, djlA*) GP108 *djlA*::ΩSpc^r^  Genevaux *et al*., 2001

IY28 (*ftsY*) BW25113 P*araCFUP*-*ftsY*, Kan^r^ Erez *et al*., 2010

**Plasmids**

pOK-HcpHA *sci1 hcp* cloned into pOK12, C-terminal HA epitope Aschtgen *et al*., 2010a

pASK-IBA37(+) Expression vector, AHT-inducible, Amp^r^ IBA technology

pIBA-TssL *sci1 tssL* cloned into pASK-IBA37(+), N-terminal FLAG epitope This study

pIBA-TssL-Lt *sci1 tssL* cloned into pASK-IBA37(+), N-terminal FLAG epitope, C-terminal lumiotag extension This study

pIBA-TssL-PG Periplasmic domain of *tagL* (residues 365-576) cloned at the C-terminus of *tssL* in pIBA-TssL This study

pIBA-TssL-ΔTM *tssL* residues 1-184 cloned into pASK-IBA37(+) (insertion of a stop codon in pIBA-TssL) This study

**Oligonucleotides**

For plasmid construction ^a, b, c^

IBA-TssL-5 tctagaaataattttgtttaactttaagaaggagatatacaaatg*gattataaagacgacgatgata*

*aa*aataaacctgttatctcccgggctgaacag

IBA-TssL-3 gatgtgatggtgatggtgatgcgatcctctttatccctgcccggtaagccgtgcc

IBA-TssL-Lt-3 gatgtgatggtgatggtgatgcgatcctctttagcaacacccggggcagcatccctgcccggtaagccgtgcc

For site-directed mutagenesis ^d^

TssL-Y184St-5 CCGTGCTGGCAGAACGATGTAATGGTTGTCATGGGGGGCGGG

TssL-Y184St-3 CCCGCCCCCCATGACAACCATTACATCGTTCTGCCAGCACGG

^a^ sequence complementary to target vector underlined.

^b^ FLAG tag coding sequence italicized.

^c^ Lumio-tag coding sequence in lower case letters.

^d^ Mismatch base underlined.
