# Supplementary material for: The C-tail anchored TssL subunit, an essential protein of the enteroaggregative Escherichia coli Sci-1 Type VI secretion system, is inserted by YidC
Source: Microbiologyopen. 2012 Mar;1(1):71–82. doi: 10.1002/mbo3.9 (PMC3426401; doi:10.1002/mbo3.9)
Supplement: Supplementary file 2 [file mbo30001-0071-SD2.ppt]

## Slide 1
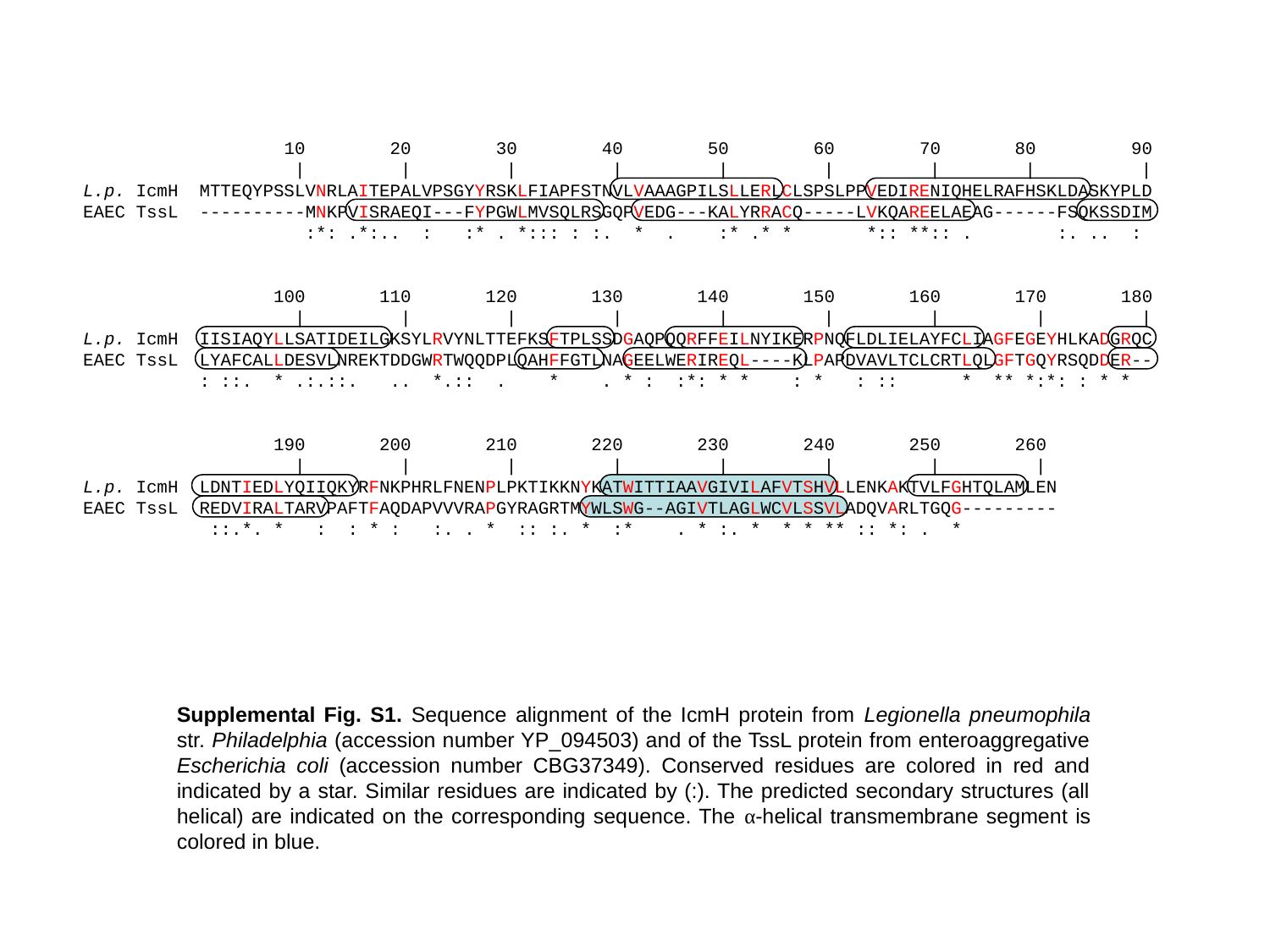

10 20 30 40 50 60 70 80 90
 | | | | | | | | |
L.p. IcmH MTTEQYPSSLVNRLAITEPALVPSGYYRSKLFIAPFSTNVLVAAAGPILSLLERLCLSPSLPPVEDIRENIQHELRAFHSKLDASKYPLD
EAEC TssL ----------MNKPVISRAEQI---FYPGWLMVSQLRSGQPVEDG---KALYRRACQ-----LVKQAREELAEAG------FSQKSSDIM
 	 :*: .*:.. : :* . *::: : :. * . :* .* * *:: **:: . :. .. :
 100 110 120 130 140 150 160 170 180
 | | | | | | | | |
L.p. IcmH IISIAQYLLSATIDEILGKSYLRVYNLTTEFKSFTPLSSDGAQPQQRFFEILNYIKERPNQFLDLIELAYFCLIAGFEGEYHLKADGRQC
EAEC TssL LYAFCALLDESVLNREKTDDGWRTWQQDPLQAHFFGTLNAGEELWERIREQL----KLPAPDVAVLTCLCRTLQLGFTGQYRSQDDER--
 : ::. * .:.::. .. *.:: . * . * : :*: * * : * : :: * ** *:*: : * *
 190 200 210 220 230 240 250 260
 | | | | | | | |
L.p. IcmH LDNTIEDLYQIIQKYRFNKPHRLFNENPLPKTIKKNYKATWITTIAAVGIVILAFVTSHVLLENKAKTVLFGHTQLAMLEN
EAEC TssL REDVIRALTARVPAFTFAQDAPVVVRAPGYRAGRTMYWLSWG--AGIVTLAGLWCVLSSVLADQVARLTGQG---------
 ::.*. * : : * : :. . * :: :. * :* . * :. * * * ** :: *: . *
Supplemental Fig. S1. Sequence alignment of the IcmH protein from Legionella pneumophila str. Philadelphia (accession number YP_094503) and of the TssL protein from enteroaggregative Escherichia coli (accession number CBG37349). Conserved residues are colored in red and indicated by a star. Similar residues are indicated by (:). The predicted secondary structures (all helical) are indicated on the corresponding sequence. The α-helical transmembrane segment is colored in blue.
